# Identification of a germline *CSPG4* variation in a family with neurofibromatosis type 1-like phenotype

**DOI:** 10.1038/s41419-021-04056-1

**Published:** 2021-08-03

**Authors:** Zhuanli Bai, Yiping Qu, Lin Shi, Xinju Li, Zhen Yang, Meiju Ji, Peng Hou

**Affiliations:** 1 Department of Plastic and Aesthetic Maxillofacial Surgery, The First Affiliated Hospital of Xi’an Jiaotong University, Xi’an, China; 2 Key Laboratory for Tumor Precision Medicine of Shaanxi Province and Department of Endocrinology, The First Affiliated Hospital of Xi’an Jiaotong University, Xi’an, China; 3 Center for Translational Medicine, The First Affiliated Hospital of Xi’an Jiaotong University, Xi’an, China

**Keywords:** Cancer genomics, Oncogenes, Cancer genetics

## Abstract

Neurofibromatosis type 1 (NF1), an autosomal dominant and multisystem disorder, is generally considered to be caused by NF1 inactivation. However, there are also numerous studies showing that Neurofibromatosis type 1-like phenotype can be caused by the abnormalities in the other genes. Through targeted parallel sequencing, whole-exome sequencing, de novo genomic sequencing, and RNA isoform sequencing, we identified a germline V2097M variation in *CSPG4* gene probably increased susceptibility to a NF1-like phenotype family. Besides, a series of in vitro functional studies revealed that this variant promoted cell proliferation by activating the MAPK/ERK signaling pathway via hindering ectodomain cleavage of CSPG4. Our data demonstrate that a germline variation in the *CSPG4* gene might be a high risk to cause NF1-like phenotype. To our knowledge, this is the first report of mutations in the *CSPG4* gene in human diseases.

## Introduction

Neurofibromatosis type 1 (NF1) is an autosomal dominant, multisystem disorder first described in 1882, and is characterized by cafe´-au-lait spots, freckling, and cutaneous neurofibromas, as well as iris hamartomas (Lisch nodules) and bone abnormalities [1, 2]. It is clear that NF1 is caused by inactivating mutations in the *NF1* gene encoding the tumor suppressor protein neurofibromin [3, 4]. Neurofibromin is a negative regulator of RAS guanosine triphosphate activity and promotes the conversion of RAS into its inactivation form under normal conditions, thereby inhibiting cell growth [5]. This process is left unhindered, leading to uncontrolled cell proliferation when there are inactivating mutations in the *NF1* gene.

Different types of mutations have been identified throughout the *NF1* gene, including compete gene deletions, insertions, and stop and splicing mutations, causing the NF1 phenotype [6, 7]. To date, although a series of variations recur in different families, no true “hotspots” have been recognized. In addition to the *NF1* gene, mutations in genes encoding some components participated in the RAS-mitogen-activated protein kinase (MAPK) pathway have also been identified in human disorders (such as Noonan syndrome, LEOPARD syndrome, Costello syndrome, and cardio-facio-cutaneous syndrome), showing some phenotypic overlap with NF1, including *PTPN11* (protein tyrosine phosphatase non-receptor type 11), *KRAS* (KRAS proto-oncogene, GTPase), *SOS1* (SOS Ras/Rac guanine nucleotide exchange factor 1), *RAF1* (Raf-1 proto-oncogene, serine/threonine kinase), *RIT1* (Ras-like without CAAX 1), *HRAS* (HRas proto-oncogene, GTPase), *BRAF* (B-Raf proto-oncogene, serine/threonine kinase), and *MEK1/2* (MAPK kinase 1/2) [8–16]. Besides, it should be noted that germline loss-of-function mutations in *SPRED1* (Sprouty-related EVH1 domain containing 1) have been identified to cause a NF1-like phenotype in 2007 [17].

In this study, we used targeted parallel sequencing to profile mutation spectrum of 504 cancer-related genes in a family with NF1-like phenotype. However, we were surprised not to find any germline abnormalities in the *NF1* gene in all affected individuals. Thus, we further performed whole-exome sequencing, de novo genomic sequencing, and RNA isoform sequencing to search for the disease-causing gene and, surprisingly, identified a germline variant in chondroitin sulfate proteoglycan 4 (*CSPG4*; also known as *NG2*) in all affected members of the kindred, but not in any unaffected members or a large cohort (*n* = 200) of healthy controls. Functional studies revealed that this variant promoted cell proliferation under certain conditions by activating the MAPK/ERK signaling pathway.

## Materials and methods

### Patients

Chinese Han family with proband and some individual members consisting of multiple cafe´-au-lait spots, axillary freckling, subcutaneous nodules, and cutaneous neurofibromas were recruited in this study (Table 1). The NF1 diagnosis was established based on the diagnostic criteria of the National Institutes of Health consensus statement of the year 1987. All study participants signed an informed consent before undergoing evaluation and genetic testing. This study was approved by the institutional review board of the First Affiliated Hospital of Xi’an Jiaotong University.

**Table 1:** Clinical features of affected individuals in family 1.

	Gender	Ages (years)	Cafe´-au-lait spots (≥6)^a^	Axillary freckling	Cutaneous neurofibromas	Plexiform neurofibroma	Optic glioma	≥2 Lisch nodules (iris hamartomas)	Osseous lesions^b^
**Family 1**									
I:2	Female	56	+	+	−	−	−	−	−
II:2	Female	25	+	+	+	−	−	−	−
II:3	Female	23	+	+	−	−	−	−	−
III:1	Female	4	+	+	−	−	−	−	−
III:2	Female	1	+	+	−	−	−	−	−
III:4	Male	2	+	+	−	−	−	−	−

^a^ver 5 mm in greatest diameter in prepubertal individuals; over 15 mm in greatest diameter in postpubertal individuals.

^b^Sphenoid dysplasia or thinning of long bone cortex with or without pseudarthrosis. –, negative; +, positive.

### DNA extraction

Genomic DNA from tumor tissue of the proband and peripheral blood samples of the family members were isolated by using a standard phenol–chloroform extraction and ethanol precipitation protocol.

### Targeted gene sequencing

A total of 504 cancer-related genes were targeted for capture and deep sequencing. Using the eArray system (Agilent, CA), the capture was designed to include all of protein coding sequences and most of the untranslated regions of these genes. In accordance with the manufacturer’s protocol, genomic DNA was fragmented by the NEBNext dsDNA Fragmentase (New England Biolabs, MA) and adaptor-ligated library was constructed using an Agilent SureSelect library kit (Agilent, CA). Targeted sequence enrichment was performed using the Agilent SureSelect Target Enrichment Kit (Agilent, CA) according to the manufacturer’s instructions. The enriched samples were sequenced via 2 × 100 paired-end sequencing using a HiSeq2000 Sequencing System (Illumina, CA). Illumina Sequencing Control v2.8, Illumina Off-Line Basecaller v1.8, and Illumina Consensus Assessment of Sequence and Variation v1.8 software (Illumina, CA) were used to produce 100 bp sequence reads.

### Whole-exome sequencing

Three micrograms of genomic DNA from peripheral blood samples of two affected individuals (F1_II:3 and F_III:1) and one healthy individual (F1_II:5) were sheared using a Covaris S1 Ultrasonicator (Covaris, MA). Adaptor-ligated libraries were constructed using Paired-End Genomic DNA kits (Illumina, CA). Exome capture was performed using a SureSeq Exome Enrichment kit (Agilent, CA) according to the manufacturer’s instructions. As mentioned above, each sample was sequenced via 2 × 100 paired-end sequencing using a HiSeq2000 Sequencing System (Illumina, CA).

### Sequencing data processing and mutation calling

SAMtools was first used to generate a binary alignment map file for affected and unaffected members from the family. The resulting reads were then aligned to the human reference genome (hg19) using the Burrows-Wheeler Aligner with default parameters. Single-nucleotide variation (SNV) calling was performed using the Genome Analysis Toolkit and VarScan programs, and the called SNV data were then combined. The functional effect of non-synonymous SNVs was assessed by the PolyPhen-2, Sorting Intolerant From Tolerant (SIFT), and MutationTaster. Non-synonymous SNVs with SIFT score of <0.05, Polyphen-2 score of >0.85, or MutationTaster score of >0.85 were considered as significant of not being benign. To sort potentially deleterious variants from benign polymorphisms, perl scripts were used to filter the SNVs against those of dbSNP135. Any SNV recorded in dbSNP135 and with a minor allele frequency of >1% in Chinese from 1000 genome database, gnomAD genomes, or TOPMed was considered as benign polymorphisms and therefore removed for subsequent analysis.

### Sanger sequencing

Thirty-two candidate variants identified by exome sequencing were validated in all members of family 1 by Sanger sequencing and the primer sequences were presented in Supplementary Table 1.

### De novo genomic sequencing and structural variant analysis

DNA libraries were prepared using the protocol provided by Oxford Nanopore Technologies (ONT). Briefly, 1 µg isolated DNA from peripheral blood samples of two affected individuals (F1_II:3 and F4_III:1) and one healthy individual (F1_II:5) was sheared to ~10 kb fragments using g-tube (Covaris), and sheared products were then purified using 0.45-fold XP beads according to Agencourt protocol. DNA fragments were then subjected to formalin-fixed paraffin-embedded DNA repair and end-repair (NEB) steps. Next, DNA was ligated to the adaptor using T4 DNA ligase. Following adaptor ligation, the products were purified by adding a 0.6-fold Agencourt XP beads and following the ONT purification protocol. The final DNA libraries were added to FLO-MIN109 flow cells and run on PromethION platform. Sniffles were then used to detect DNA structural variants.

### RNA isoform sequencing and data analysis

One microgram of total RNA from peripheral blood samples of two affected individuals (F1_II:3 and F1_III:1) and one healthy individual (F1_II:5) was prepared for cDNA libraries using protocol provided by ONT. In brief, SuperScript IV First-Strand Synthesis System (Invitrogen) was used for full-length mRNA reverse transcription and following cDNA PCR for 14 circles with LongAmp Tag (NEB). Agencourt XP beads were used for DNA purification according to ONT protocol. The final cDNA libraries were added to FLO-MIN109 flow cells and were run on PromethION platform at Biomarker Technology Company (Beijing, China).

### Sodium bisulfite treatment and MSP

The protocols of sodium bisulfite treatment and methylation-specific PCR (MSP) were performed as described previously [18]. The primer sequences for MSP assay were summarized in Supplementary Table 2.

### Immunohistochemistry

Immunohistochemistry was performed to investigate the expression levels of S100 protein in tumor tissue of the proband from family 1. Briefly, paraffin-embedded tissue sections (5 μm) were deparaffinized and rehydrated in xylene and degradation alcohol. After antigen retrieval using microwave heating for 15 min, the slides were washed and incubated with anti-S100 antibody (Abcam, Inc.) overnight at 4 °C. Immunodetection was performed with the Streptavidin-Peroxidase system (ZSGB-bio, Beijing, China) according the manufacturer’s protocol, followed by reaction with diaminobenzidine and counterstaining with hematoxylin.

### Site-directed mutagenesis

The V2097M mutation in the *CSPG4* gene were generated by site-directed mutagenesis of wild-type human *CSPG4* cDNA clone (Origene, MD), which was cloned into a eukaryotic expression vector (pCMV6) using QuikChange Site-directed mutagenesis kit from Stratagene (Santa Clara, CA). The following primers were used for site-directed mutagenesis: 5′-CAT GGC CGC GTG GTC CGC ATG CCC CGA GCC AGG AC-3′ (forward) and 5′-GTC CTG GCT CGG GGC ATG CGG ACC ACG CGG CCA TG-3′ (reverse).

### Cell line and transfection

Glioma cell line U251 was purchased from American Type Culture Collection and was authenticated by short tandem repeat profiling. Cells were routinely cultured at 37 °C in Dulbecco’s modified Eagle medium (DMEM) with 10% fetal bovine serum (FBS), and were transfected with the indicated constructs at 70% confluence using Lipofectamine 2000 (Life Technologies, MD) according to the manufacturer’s instructions.

### Cell proliferation assay

Cell proliferation was monitored in real-time using the iCELLigence system electronic microtiter plate. Cells (20,000/well) were seeded and cultured in 16-well plates. The impedance value of each well was automatically monitored by the iCELLigence system for a duration of 120 h and was expressed as a cell index (CI) value. After a 24 h culture, the medium was then changed to DMEM medium with 0.5% FBS or DMEM medium with 20 ng/mL vascular endothelial growth factor (VEGF). In some experiments, phorbol 12-myristate 13-acetate (PMA; Sigma, MO) and ectopic expression of ADAM metallopeptidase domain 10 (ADAM10) were used to promote the proteolysis of CSPG4 and amplify the MAPK/ERK signaling. Data were then normalized at 26 h. Normalized CI is calculated by dividing CI at the normalized time into the original CI. The rate of cell proliferation was determined by calculating the slope of the line between two given time points.

### Western blot analysis

The protocol was previously described [19] and antibody information was presented in Supplementary Table 3.

### Statistical analysis

Values were shown as mean ± SEM. An unpaired Student’s *t*-test was used for comparison between groups and analysis of variance for multiple group comparison in Graphpad Prism. *P* < 0.05 was considered to be statistically significant.

## Results and discussion

### Case reports

**Fig. 1:**
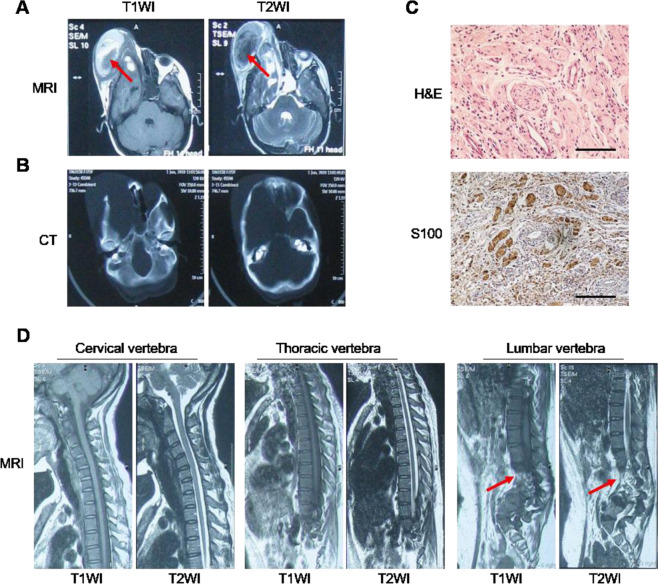
Clinical and imaging features of the proband of family 1. A large mass (indicated by arrows) grew outward from the orbit of the eye in the right temporal lobe, with mixed signals with hemorrhage in magnetic resonance imaging (MRI, **A**) and computed tomography (CT, **B**) imaging. **C** Immunohistochemical analysis showing the involvement of S100-positive Schwann cells in the tumor tissue. Scale bar: 200 µm. **D** The MRI of cervical vertebra, thoracic vertebra, and lumbar vertebra demonstrating lumbar scoliosis (indicated by arrows).

**Fig. 2:**
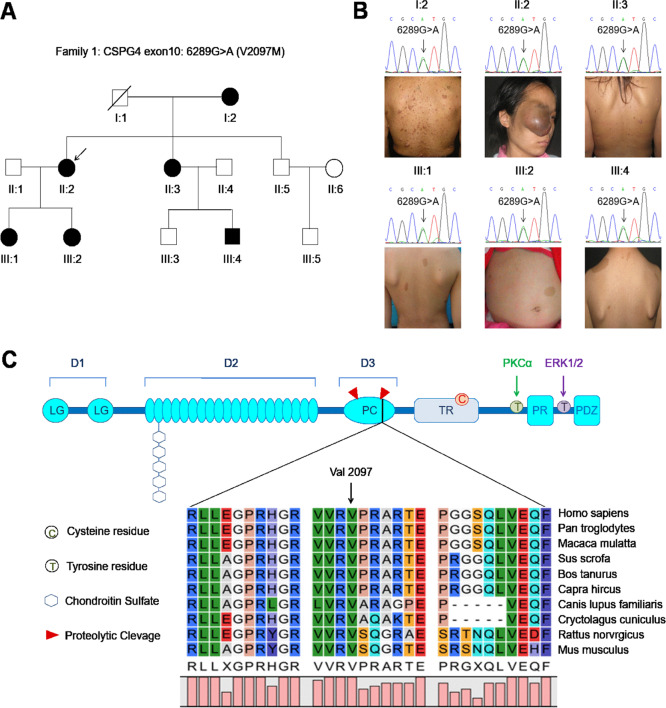
Pedigrees and clinical photographs of family 1. **A** The pedigrees of family 1. Squares and circles indicate males and females, respectively. Open symbols indicate unaffected individuals, filled symbols indicate affected individuals, and symbols with a slash indicate deceased family members; the arrow points to the proband (II:2). **B** Clinical phenotypes of six affected individuals from family 1. Sanger sequencing was used to validate CSPG4 V2097M variation at base 6289 in affected members of family 1. **C** The protein domain architecture of CSPG4 and conservation of the V2097 position across species. CSPG4 is composed of three major structural components: the extracellular domain, the transmembrane region, and the cytoplasmic C-terminal domain (CTD). The extracellular domain contains an N-terminal globular subdomain (D1) consisting of laminin G-type regions (LG) and disulfide bonds. The D2 subdomain consists of 15 CSPG repeats. The D3 globular subdomain contains sites for proteolytic cleavage by matrix metalloproteinases (MMPs) or other proteases. The transmembrane region of CSPG4 contains a cysteine residue (C) at position 2230 that may play a role in CSPG4 membrane localization, although this is yet to be elucidated. The CTD contains tyrosine residues (T) that serve as phosphoacceptor sites for PKCa and ERK1/2. The proline-rich region (PR) may comprise a non-canonical SH3 protein interaction domain and the C terminus contains a four-residue PDZ domain-binding motif (PDZ) that is responsible for interactions with various PDZ domain-containing binding partners.

As shown in Table 1, the affected individuals from family 1 fulfilled the NIH diagnostic criteria for NF1. However, some typical features were systematically absent, such as Lisch nodules in the iris and central nervous system tumors [20, 21]. The proband of family 1 (Patient II:2) is a 25-year-old woman having signs of multiple CALMs (Cafe-au-lait macules), freckling of axillary and cutaneous neurofibromas that manifested in early adolescence (Table 1). During pregnancy, the plexiform neurofibromas in her right orbital grew dramatically and was partially resected later to relieve the symptoms (Fig. 1A, B). The computed tomography images showed enlargement of the right middle cranial fossa and right orbit, and compression of the bone. The pathological diagnosis of neurofibroma was validated by both light microscopy analysis (hematoxylin and eosin staining) and immunohistochemical analysis (S100 staining for Schwann cells) in the dissection specimens (Fig. 1C). The magnetic resonance images of proband’s cervical, thoracic, and lumbar vertebra also showed skeletal abnormalities such as lumbar scoliosis. However, no enlargement and abnormal signals were found in the spinal cord and no space-occupying lesions were found in the vertebral canal (Fig. 1D). Her mother, young sister, two daughters, and nephew were all affected at the time of the initial evaluation (Table 1 and Fig. 2A). Moreover, the three children in this family were all born with cafe´-au-lait spots and the proband’s mother and young sister exhibit more verruca on the skin with years. Besides, none of these family members had a history of other primary cancers and no other benign tumors were detected in the kindred.

### Identification of *CSPG4* as a susceptibility gene causing NF1-like phenotype

To identify the susceptibility genes in this family, we performed targeted massively parallel sequencing of 504 genes, which are commonly mutated in human cancers. Through systematic analysis, however, except for the proband’s tumor tissue, we were surprised not to find any mutations in the *NF1* or *SPRED1* gene in peripheral blood DNA in all members of family 1 (Supplementary Table 4 and Supplementary Fig. S2).

To find out the pathogenic gene in the family, we next performed whole-exome sequencing using peripheral blood DNA from two affected family members (F1_II:3 and F1_III:1) and healthy member (F1_II:5), and identified specific 78 single-nucleotide variants that were present only in the two affected family members (Supplementary Table 5). Similarly, we did not find any *NF1* mutations in these two affected family members, further supporting the above conclusion. Next, by using filtering criteria and bioinformatics analysis (as described in “Materials and Methods”), 32 candidate variants were left for further validation using Sanger sequencing in the whole family members. Finally, we selected *CSPG4* as a candidate gene, because a G → A substitution at base 6289 in exon 3 was only found in all these six affected members, but not in any three unaffected members (Fig. 2B and Supplementary Fig. S3) or a cohort of 200 healthy people randomly selected from the Physical Examination Center of the First Affiliated Hospital of Xi’an Jiaotong University (data not shown). This variant results an amino acid change from valine to methionine at position 2097 (V2097M). All affected family members were heterozygous for this variant in peripheral blood DNA (Fig. 2B).

CSPG4, as a transmembrane proteoglycan, is strongly associated with malignant progression and poor prognosis in many cancers by activating some major pathways as a co-receptor in partnership with certain receptor tyrosine kinases (RTKs) [22, 23]. The CSPG4 core protein consists of three main structural domains: a large extracellular domain, a 25-amino acid transmembrane region, and a 75-amino acid cytoplasmic domain. Moreover, its extracellular domain also contains three subdomains termed D1–3 (Fig. 2C upper panel) [22]. This V2097M variant is located in the D3 domain where it contains a number of putative proteolytic cleavage sites and is a highly conserved site not only in human but also in other species (Fig. 2C lower panel).

### CSPG4 V2097M variant promotes cell proliferation by activating the MAPK/ERK signaling pathway via hindering its ectodomain cleavage

**Fig. 3:**
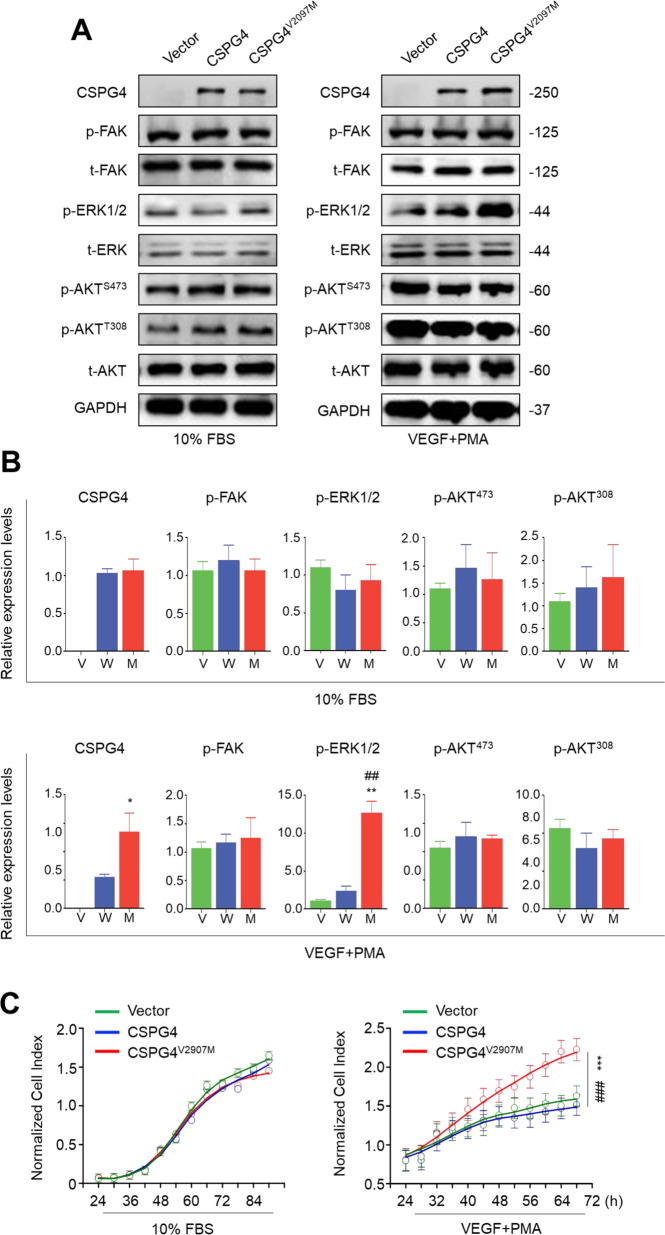
Functional characterization of CSPG4 V2097M variation. **A** Western blot analysis in U251 cells transfected with vector, wild-type, and V2097M CSPG4 under the indicated conditions. Antibodies against phospho-FAK (p-FAK), total FAK (t-FAK), phospho-AKT (p-AKT), total AKT (t-AKT), phospho-ERK (p-ERK), and total ERK1/2 (t-ERK) were used to test the effect of the above treatments on the activities of CSPG4-regulated pathways. GAPDH was used as a loading control. **B** The quantitative illustration of the levels of the indicated proteins using densitometry to measure the density of the corresponding bands on the western blotting shown in **a**. V, Vector; W, wild-type CSPG4; M, V2097M CSPG4. **C** Cell proliferation assay in U251 cells transfected with different constructs under the indicated conditions. Statistically significant differences were indicated: ***P < 0.001 for comparison with vector; ^###^*P* < 0.001 for comparison with wild-type CSPG4.

It is proved that CSPG4 could regulate the activities of some important signaling pathways such as RAS/MAPK signaling as a co-receptor in partnership with certain RTKs [22, 23]. To determine the biological role of this V2097M variant, we constructed expression plasmids for wild-type and V2097M CSPG4, and ectopically expressed them in a CSPG4-negative glioma cell line U251 under different culturing conditions. Next, we tested their effect on the activities of CSPG4-regulated pathways such as focal adhesion kinase (FAK), phosphatidylinositol 3-kinase/AKT (AKT serine/threonine kinase), and MAPK/ERK pathways by western blot analysis. As shown in Fig. 3A (left panel), compared to vector and wild-type CSPG4, ectopic expression of V2097M CSPG4 almost did not affect the phosphorylation of FAK, ERK, and AKT under normal culturing condition (DMEM medium containing 10% FBS).

**Fig. 4:**
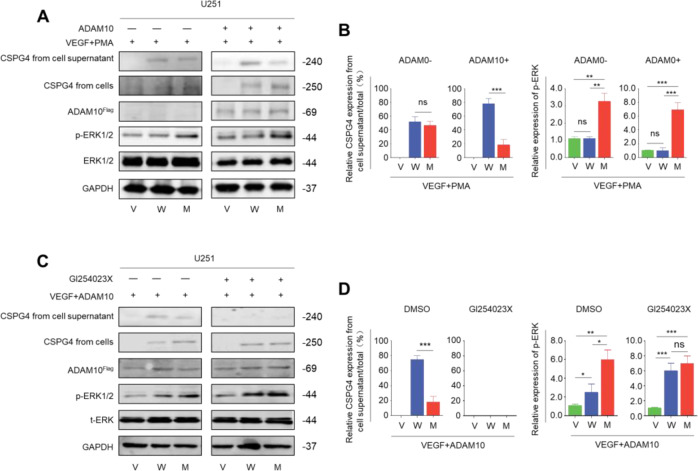
V2097M variation hinders ectodomain cleavage of CSPG4. **A** Western blot analysis in U251 cells transfected with vector, wild-type, and V2097M CSPG4. Antibodies against CSPG4, ADAM10, phospho-ERK (p-ERK), and total ERK1/2 (t-ERK) were used to test the effect of the above treatments on the activity of MAPK/ERK pathway and ectodomain cleavage of CSPG4. GAPDH was used as a loading control. **B** The quantitative illustration of the levels of the indicated proteins using densitometry to measure the density of the corresponding bands on the western blotting shown in **a**. **C** U251 cells transfected with vector, wild-type, and V2097M CSPG4 were treated with 20 nM GI254023X or DMSO. Western blot analysis was then performed to determine the effect of the above treatments on the activity of MAPK/ERK pathway and ectodomain cleavage of CSPG4. GAPDH was used as a loading control. **D** The quantitative illustration of the levels of the indicated proteins using densitometry to measure the density of the corresponding bands on the western blotting shown in **c**. V, vector; W, wild-type CSPG4; M, V2097M CSPG4. Statistically significant differences were indicated: ns, no significance; **P < 0.01; ***P < 0.001.

Considering that this variant is close to a putative proteolytic cleavage site in the D3 domain, thus we speculate that it may impair ectodomain cleavage of CSPG4 [24, 25]. To test our hypothesis, U251 cells whether expressing wild-type and V2097M CSPG4 were cultured using the DMEM medium containing 20 ng/mL VEGF. Meanwhile, PMA was added to the above medium not only to amplify the MAPK/ERK cascade but also to promote the cleavage of CSPG4 by some proteases [26–29]. This medium was termed the PMA-stimulated medium. As shown in Fig. 3A (right panel) and Fig. 3B, mutant CSPG4 levels were clearly elevated compared to wild-type CSPG4 under such conditions when they had a very similar expression efficiency (data not shown). These results further support the involvement of this variant in regulating ectodomain cleavage of CSPG4. Besides, compared to wild-type CSPG4, mutant CSPG4 activated the MAPK/ERK pathway, characterized by increased phosphorylation of ERK, whereas it almost did not affect phosphorylation of FAK and AKT in the PMA-stimulated medium (Fig. 3A, B). Correspondingly, compared to vector and wild-type CSPG4, mutant CSPG4 did not affect cell proliferation under normal culturing condition (Fig. 3C left panel), whereas significantly promoted cell proliferation under the PMA-stimulated medium (Fig. 3C right panel). There is evidence showing that some α-secretases, such as ADAM10, are responsible for the cleavage of CSPG4 [30, 31], as supported by our data that ectopic expression of ADAM10 in U251 cells promoted ectodomain cleavage of wild-type CSPG4 compared to mutant CSPG4 under DMEM medium containing 20 ng/mL VEGF and PMA (Fig. 4A, B). Besides, we expectedly found that ectopic expression of ADAM10 further increased ERK phosphorylation relative to the vector (Fig. 4A, B). These effects could be effectively reversed by ADAM10 inhibitor GI254023X (Fig. 4C, D), further supporting the above conclusions.

Considering that gene promoter hypermethylation, deletion, or aberrant splicing may also cause inactivation of tumor suppressor genes including *NF1* [32–35], thus we further investigated promoter methylation of *NF1* gene in leukocyte DNA of six affected individuals and three unaffected individuals in family 1 using the MSP assay. However, we did not find *NF1* methylation in these samples (Supplementary Fig. S4). Besides, we also did not find structural variants (such as chromosomal rearrangements) and splicing alterations in *NF1* (or *CSPG4*) gene of this family in two affected individuals and one healthy individual by using de novo genomic sequencing (Date not shown) and RNA isoform sequencing (Supplementary Table 6). This was also supported by western blot analysis of NF1 proteins in the proband’s skin tissue and a normal healthy skin tissue (Supplementary Fig. S5). Taken together, our data demonstrate that this variant causes a NF1-like phenotype by hindering ectodomain cleavage of CSPG4 and subsequently activating the MAPK/ERK signaling.

**Fig. 5:**
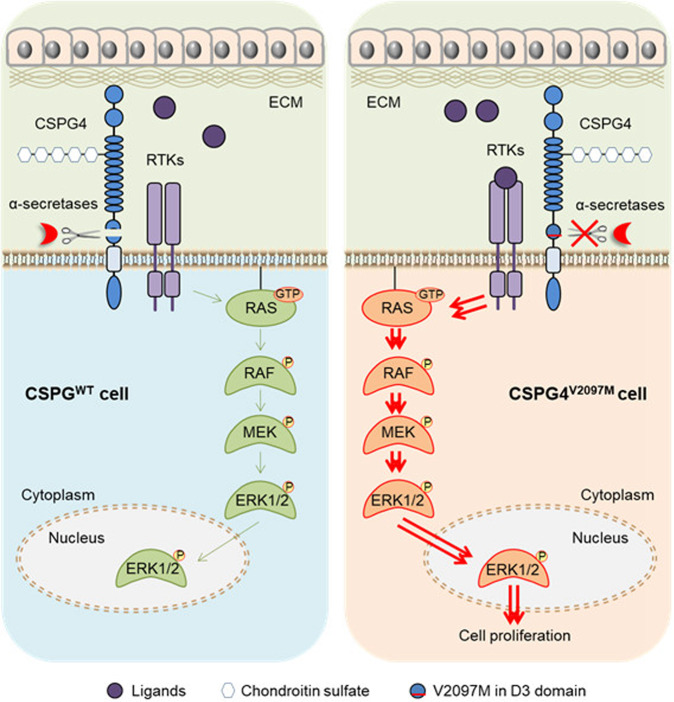
Working model for the mechanism of CSPG4 V2097M variation promoting cell proliferation. Normally, CSPG4 acts as a co-receptor to modulate the activity of RAS/MAPK signaling by cooperating with some RTKs, whereas α-secretases, such as ADAM10, are responsible for the shedding and processing of the CSPG4 proteoglycan, thereby controlling the activity of RAS/MAPK cascade at relatively low levels in normal cells. However, a V2097M variation located at the D3 domain of CSPG4 is close to the putative proteolytic cleavage sites, hindering ectodomain cleavage of CSPG4. This will result in a sustained activation of MAPK/ERK signaling, thereby promoting cell proliferation.

Given the above, we proposed a model to illustrate the mechanism of this variation causing a NF1-like phenotype (Fig. 5). In normal cells, CSPG4 as a co-receptor can modulate the activity of MAPK signaling through cooperating with some RTKs, whereas α-secretases, such as ADAM10, will maintain a relatively low activity of this pathway by promoting the shedding and processing of the CSPG4 proteoglycan. However, a V2097M variation located at the D3 domain of CSPG4, which is close to the putative proteolytic cleavage sites, sustainably activates the MAPK/ERK signaling by hindering ectodomain cleavage of CSPG4, thereby promoting cell proliferation.

## Conclusions

In summary, we identify a germline V2097M variation in *CSPG4* gene in individuals with a mild NF1-like phenotype and demonstrate that this variation can promote cell proliferation by enhancing the MAPK/ERK signaling via hindering ectodomain cleavage of CSPG4. However, the main limitation of this study is the lack of in vivo evidence to demonstrate this variation causing a NF1-like phenotype. In addition, two questions come to mind that will be addressed in future studies. First, should members of affected families, who are carriers of this variant, be strictly followed up or treated as NF1 patients? Second, does this variant confer a predisposition to other diseases such as malignancies?

## Supplementary information

Supplementary Figures

Supplementary Table S1

Supplementary Table S2

Supplementary Table S3

Supplementary Table S4

Supplementary Table S5

Supplementary Table S6

## Data Availability

The datasets used and/or analyzed during the current study are available from the corresponding author on reasonable request.
