# Supplementary material for: Identification of a germline CSPG4 variation in a family with neurofibromatosis type 1-like phenotype
Source: Cell Death Dis. 2021 Aug 3;12(8):765. doi: 10.1038/s41419-021-04056-1 (PMC8333038; doi:10.1038/s41419-021-04056-1)
Supplement: Supplementary file 1 — Supplementary Figures [file 41419_2021_4056_MOESM1_ESM.docx]

**Supplementary Figure legends**

**Figure S1. The validation of *NF1* status in family 1.** All family members except for the proband’s tumor tissue were demonstrated not to carry any mutations in the NF1 gene. The proband’s tumor tissue was found with a very low frequency of somatic NF1 mutation (G→A substitution at base 1642 in exon 14), resulting in a change in the amino acid from glutamine to glutamic at position 41 (G41E).

**Figure S2.** Three unaffected members of family 1 were demonstrated to carry wild-type CSPG4 by Sanger sequencing.

**Figure S3. Methylation status of *NF1* promoter in family 1.** MSP assay was performed to assess promoter methylation of NF1 gene in 6 affected individuals and 3 unaffected individuals from family 1. In vitro methylated DNA was used as a positive control for methylated gene (MP), bisulfite-modified normal leukocyte DNA as a positive control for unmethylated gene (UP), and H2O as a blank control to confirm the specificity of MSP. Mk, DNA marker; M, methylated gene; U, unmethylated gene.

**Figure S4.** Western blot analysis was performed to evaluate NF1 expression and gene splicing in the proband’s skin tissue and a normal healthy skin tissue. GAPDH was used as an internal reference.

##
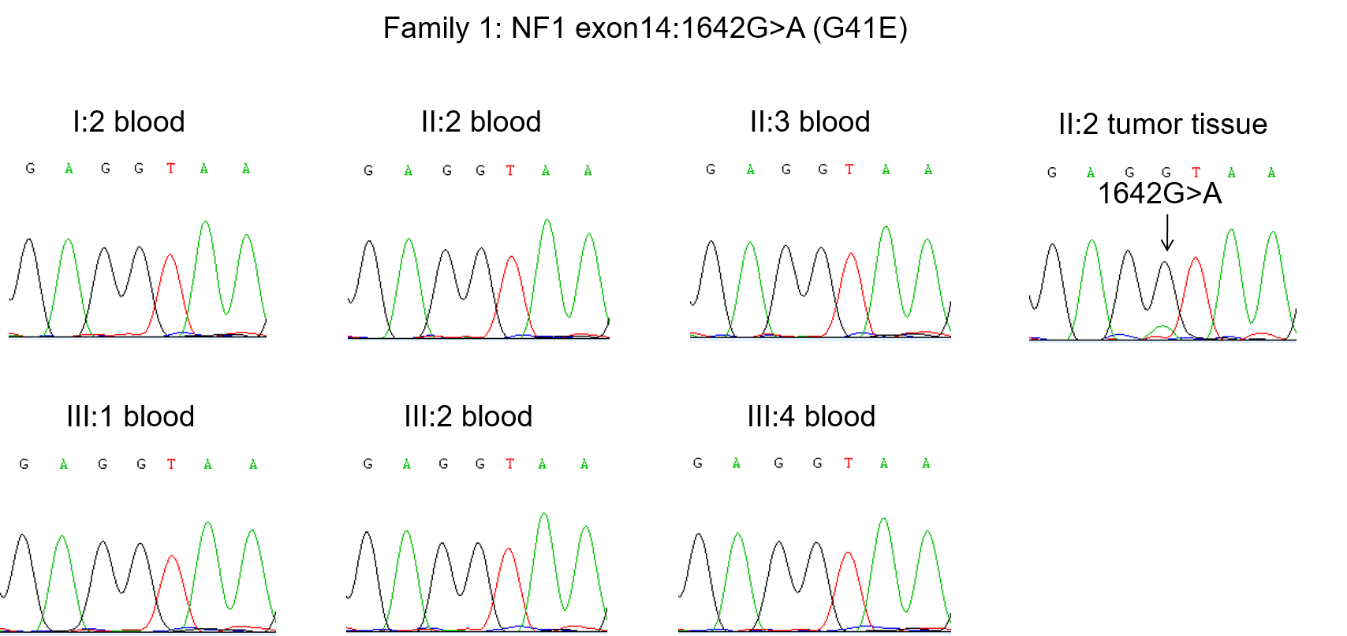


## Figure S1

##
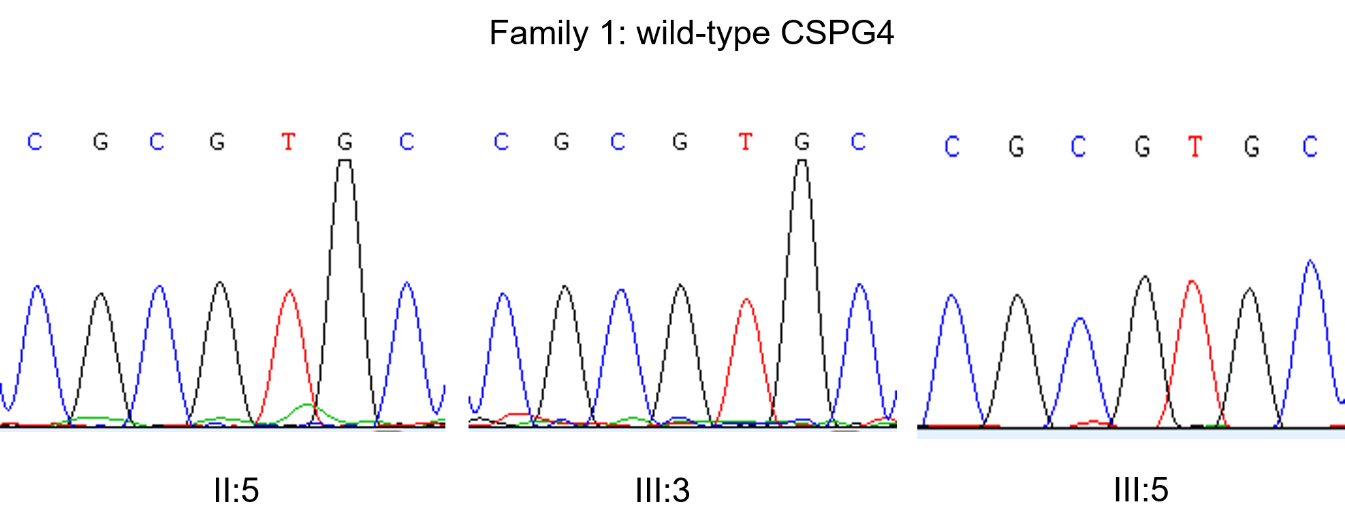


**Figure S2**

##
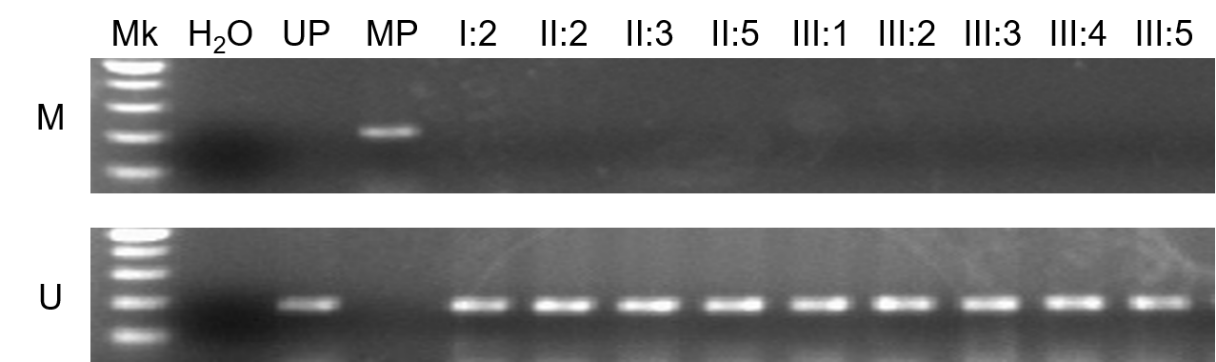


-174

-174

## Figure S3

##
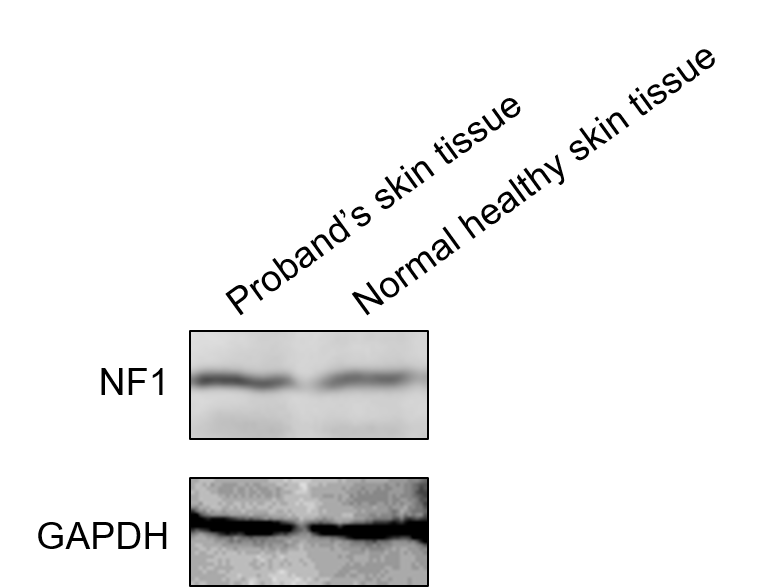


-320kd

-37kd

## Figure S4
