# Supplementary material for: Identification of a germline CSPG4 variation in a family with neurofibromatosis type 1-like phenotype
Source: Cell Death Dis. 2021 Aug 3;12(8):765. doi: 10.1038/s41419-021-04056-1 (PMC8333038; doi:10.1038/s41419-021-04056-1)
Supplement: Supplementary file 2 — Supplementary Table S1 [file 41419_2021_4056_MOESM2_ESM.docx]

**Table S1.** The primers used for PCR and Sanger sequencing

| **Num** | **Genes** | **Forward primer (5'→3')** | **Reverse primer (5'→3')** |
| --- | --- | --- | --- |
|  | ***NF1-family 1*** | AATCCTTGGAATTAACAGC | CATAATCATTACTTGACATACC |
| **1** | ***APLP2*** | TCCATCAAGCAGCAGGTAG | CCCACTCTTCCCAATCTCC |
| **2** | ***ALPP*** | GGACGGGAAGAATCTGGTGC | TCTCGGTGGATCTCGTATTTCA |
| **3** | ***BCLAF1*** | ACTGGCAGGCCCTAGCAT | CAAAGGTTCCTCGTGGTC |
| **4** | ***CCDC9*** | GACTGTATTTTCCCCATCTC | GGACACACAGGACAAATG |
| **5** | ***CSPG4*** | TGGTCCGGCACAAGAAGA | GCGACACCATCACCAGGTAG |
| **6** | ***CSPG4*** | CTTCACCAACTTCTCCTC | CATTGTAAGGCTCAGTGG |
| **7** | ***CYP2A13*** | CTCCCACCCCACTCCCTCT | CCACGCCAAAACCCCTTAG |
| **8** | ***DCAF12L2*** | CTGGGCACCCTCAACAAG | GGCATATACTGGGAGACC |
| **9** | ***DNAH9*** | ACCCACCTACTCTACCAC | GTTACCACTTCCATTTCACAG |
| **10** | ***DNAH9*** | GGGTATTAGGCTCCAGATG | GAAGACAGGATGGCTCAG |
| **11** | ***DUSP16*** | CTGTGCCGACTCTGCTAC | ATGCTGGCTGAATATGAAA |
| **12** | ***EPPK1*** | GAACTCCTCAGCTCAGCCATAA | CAGCACGCCCTTCCACAT |
| **13** | ***FCGRT*** | ATCAGACACTTGGTGCTGGAA | AGCCCACTCCTCATCCTTCT |
| **14** | ***FGL1*** | CGGGTCAAACAGCAACAG | AGGCAGGAGAATGGTGTG |
| **15** | ***FMNL2*** | GGTCCAGTAACACCACCTATG | AAAACCACTGTGGGTGAAGA |
| **16** | ***HMHA1*** | CGGACTCGGACCTAGAGGAG | TGCAGCACGTTGTTGGACT |
| **17** | ***IRX1*** | CAGCAGCCGACGTTCTCAAGC | GGAATGCGCTGTTGGTCCAGTT |
| **18** | ***KLK5*** | GAGTTGAGGATGGTTTGG | GAGAAGTGGAGAAAGATGG |
| **19** | ***MEIS1*** | TCTGTTCCTCTTGCTCCTC | CGACTACGTGCTGGCTACT |
| **20** | ***MYH2*** | TGAAAGAATGAAAGGTGGCT | GCAGAAGATGCTGGAGGA |
| **21** | ***NEFM*** | CGTGTCCTCCTCCTATAAG | GATGTCTTCCTCCAGGTG |
| **22** | ***NR1H2*** | CCTACAGGCGTCCTTTCTGA | GCTGTTAGCTGGACACCCTC |
| **23** | ***PABPC3*** | CCAGGTTGTTAATCTTTATG | AACTGCTTTAGTGGCTTC |
| **24** | ***POLR3B*** | GCACGGTGTTAAGCACATA | AATCACCTGGTCGGCAATC |
| **25** | ***ROBO4*** | GGTTAGGTTATGTTTAGGG | AAGGACAGTGGAGTTATC |
| **26** | ***RP1L1*** | GAAGAGGCGGTGCAGTTAG | GCCCTTCTCCTCCTGTTTC |
| **27** | ***SUSD2*** | GCACCTGCTGCTTGGATTT | CGAGGGTCTGGATGCTGTC |
| **28** | ***SYF2*** | ATGTCCTGCCTGACCCTC | TAGTTCCCACTCCAAACG |
| **29** | ***TEMEM222*** | AGCCAAGCAAGTGTCCAG | GCATCTGTTCCAACTGTCC |
| **30** | ***TIRAP*** | TGGAGCAAAGACTATGAC | GAACTACTAGGCTACAGG |
| **31** | ***ZNF703*** | CTATTGCTTGGGAGGTTACCA | AGTGGCTCTTGCCATAGG |
| **32** | ***ZNF747*** | TGTACCGGGACGTGATGCG | TCCTCCACCCAGGAGATGAGC |
