# Supplementary material for: Identification of a germline CSPG4 variation in a family with neurofibromatosis type 1-like phenotype
Source: Cell Death Dis. 2021 Aug 3;12(8):765. doi: 10.1038/s41419-021-04056-1 (PMC8333038; doi:10.1038/s41419-021-04056-1)
Supplement: Supplementary file 3 — Supplementary Table S2 [file 41419_2021_4056_MOESM3_ESM.docx]

Table S2. Genomic variants of the family 1 member

| **Num** | | **Gene** | **I:2** | **II:2** | **II:3** | **II:5** | **III:1** | **III:2** | **III:3** | **III:4** | **III:5** |
| --- | --- | --- | --- | --- | --- | --- | --- | --- | --- | --- | --- |
| **1** | ***APLP2 (1972A>G)*** | | A/A | A/G | A/G | A/A | A/G | A/G | A/A | A/G | A/A |
| **2** | ***ALPP (865+8 C>T)*** | | C/T | C/T | C/T | C/C | C/T | C/T | C/C | C/C | C/C |
| **3** | ***BCLAF1 (2249 G>T)*** | | G/G | G/T | G/T | G/G | G/T | G/T | G/G | G/T | G/G |
| **4** | ***CCDC9 (452G>A)*** | | G/A | G/A | G/A | G/G | G/A | G/G | G/G | G/A | G/G |
| **5** | ***CSPG4 (1321G>A)*** | | C/C | C/T | C/T | C/C | C/T | C/C | C/C | C/C | C/C |
| **6** | ***CSPG4 (6289G>A)*** | | C/T | C/T | C/T | C/C | C/T | C/T | C/C | C/T | C/C |
| **7** | ***CYP2A13 (350 C>T)*** | | C/C | C/T | C/T | C/C | C/T | C/T | C/C | C/T | C/C |
| **8** | ***DCAF12L2 (414C>A)*** | | G/G | G/T | G/T | G/G | G/T | G/T | G/G | G/T | G/G |
| **9** | ***DNAH9 (5525G>A)*** | | G/G | G/A | G/A | G/G | G/A | G/G | G/G | G/G | G/G |
| **10** | ***DNAH9 (1010C>T)*** | | C/C | C/T | C/T | C/C | C/T | C/C | C/C | C/C | C/C |
| **11** | ***DUSP16 (1091_1099del)*** | | GCACGCTGG/GCACGCTGG | GCACGCTGG/- | GCACGCTGG/- | GCACGCTGG/GCACGCTGG | GCACGCTGG/- | GCACGCTGG/GCACGCTGG | GCACGCTGG/- | GCACGCTGG/GCACGCTGG | GCACGCTGG/GCACGCTGG |
| **12** | ***EPPK1 (6716G>A)*** | | C/C | C/T | C/T | C/C | C/T | C/C | C/C | C/C | C/C |
| **13** | ***FCGRT (916 G>A)*** | | G/A | G/A | G/A | G/G | G/A | G/A | G/G | G/G | G/A |
| **14** | ***FGL1 (206A>G)*** | | T/T | T/C | T/C | T/T | T/C | T/T | T/C | T/T | T/T |
| **15** | ***FMNL2 (1671_1672insCCA)*** | | -/- | -/CCA | -/CCA | -/- | -/CCA | -/CCA | -/- | -/- | -/- |
| **16** | ***HMHA1(2912_2913insGGACGA)*** | | -/- | -/GGACGA | -/GGACGA | -/- | -/GGACGA | -/GGACGA | -/- | -/- | -/- |
| **17** | ***IRX1 (1039T>G)*** | | T/T | T/G | T/G | T/T | T/G | T/T | T/T | T/T | T/T |
| **18** | ***KLK5 (497C>G)*** | | G/G | G/C | G/C | G/G | G/C | G/G | G/G | G/C | G/G |
| **19** | ***MEIS (298A>C)*** | | A/A | A/C | A/C | A/A | A/C | A/A | A/A | A/A | A/A |
| **20** | ***MYH2 (4258 C>T)*** | | C/T | C/T | C/T | C/C | C/T | C/T | C/C | C/C | C/T |
| **21** | ***NEFM (436C>G)*** | | C/G | C/G | C/G | C/C | C/G | C/G | C/C | C/C | C/C |
| **22** | ***NR1H2 (225_226insCAG)*** | | -/CAG | -/CAG | -/CAG | -/- | -/CAG | -/- | -/- | -/CAG | -/- |
| **23** | ***PABPC3 (975_979del)*** | | TATGA/TATGA | TATGA/- | TATGA/- | TATGA/TATGA | TATGA/- | TATGA/TATGA | TATGA/TATGA | TATGA/TATGA | TATGA/TATGA |
| **24** | ***POIR3B (1102C>T)*** | | C/T | C/T | C/T | C/C | C/T | C/C | C/C | C/C | C/T |
| **25** | ***ROBO4 (2887G>A)*** | | C/C | C/T | C/T | C/C | C/T | C/T | C/C | C/T | C/C |
| **26** | ***RP1L1 (4003G>A)*** | | C/C | C/T | C/T | C/C | C/T | C/T | C/C | C/C | C/C |
| **27** | ***SUSD2 (271G>A)*** | | G/A | G/A | G/A | G/G | G/A | G/A | G/G | G/A | G/A |
| **28** | ***SYF2 (214A>G)*** | | T/C | T/C | T/C | T/T | T/C | T/C | T/C | T/C | T/C |
| **29** | ***TMEM222 (421T>A)*** | | T/A | T/A | T/A | T/T | T/A | T/A | T/A | T/A | T/T |
| **30** | ***TIRAP (601G>C)*** | | G/G | G/C | G/C | G/G | G/C | G/C | G/G | G/C | G/G |
| **31** | ***ZNF703 (1253A>C)*** | | A/A | A/C | A/C | A/A | A/C | A/C | A/A | A/C | A/A |
| **32** | ***ZNF747 (236G>A)*** | | C/T | C/T | C/T | C/C | C/T | C/C | C/C | C/T | C/C |
