# Supplementary material for: Identification of a germline CSPG4 variation in a family with neurofibromatosis type 1-like phenotype
Source: Cell Death Dis. 2021 Aug 3;12(8):765. doi: 10.1038/s41419-021-04056-1 (PMC8333038; doi:10.1038/s41419-021-04056-1)
Supplement: Supplementary file 4 — Supplementary Table S3 [file 41419_2021_4056_MOESM4_ESM.docx]

**Table S3.** The primer sequences used in this study for MSP assay

| **Gene** | **Allele** | **Forward primer (5'→3')** | **Reverse primer (5'→3')** |
| --- | --- | --- | --- |
| *NF1* | M | GTGAGGGACGTTCGTTAGAC | AACGCGAACGAACTAAAAATT |
|  | U | GGTGAGGGATGTTTGTTAGAT | AAACACAAACAAACTAAAAATT |

M, methylated gene; U, unmethylated gene
