# Supplementary material for: Identification of a germline CSPG4 variation in a family with neurofibromatosis type 1-like phenotype
Source: Cell Death Dis. 2021 Aug 3;12(8):765. doi: 10.1038/s41419-021-04056-1 (PMC8333038; doi:10.1038/s41419-021-04056-1)
Supplement: Supplementary file 5 — Supplementary Table S4 [file 41419_2021_4056_MOESM5_ESM.docx]

**Table S4**. The antibodies used in this study

| **Antibodies** | **Catalog#** | **Source** |
| --- | --- | --- |
| anti-CSPG4 | ab129051 | (Abcam) |
| anti-FAK | #3285 | (Cell Signaling) |
| anti-p-FAK | #3283 | (Cell Signaling) |
| anti-Flag | #8146 | (Cell Signaling) |
| anti-p-ERK1/2 | #4370 | (Cell Signaling) |
| anti-t-ERK | #9102 | (Cell Signaling) |
| anti-p-AKT473 | BS4007 | (Bioworld) |
| anti-p-AKT308 | BS4647 | (Bioworld) |
| anti-t-AKT | BS1379 | (Bioworld) |
| anti-NF1 | orb214303 | (biorbyt) |
| anti-GAPDH | AP0063 | (Bioworld) |
